# Reliability and Validity of the Load-Velocity Relationship for Predicting the 1 RM in the Bulgarian Split Squat Exercise

**DOI:** 10.5114/jhk/205096

**Published:** 2025-11-19

**Authors:** Hao Yan, Haiting Zhai, Tingting Wang, Duchun Wang, Hongwen Wei

**Affiliations:** 1 School of Strength and Conditioning Training, Beijing Sport University, Beijing, China.; 2 School of Sports Coaching, Beijing Sport University, Beijing, China.; 3 Department of Chinese Table Tennis Association, Beijing, China.

**Keywords:** relative load, strength training, barbell velocity

## Abstract

This study aimed to compare the reliability and validity of various prediction models based on load-velocity relationships for predicting the one-repetition maximum (1 RM) in Bulgarian split squat (BSS) exercises. Twenty-seven resistance-trained men participated in the study, completing both a 1-RM test and a progressive loading test to determine the 1 RM value, along with the mean velocity (MV) and peak velocity (PV) for each load. Load-velocity relationships were constructed using linear and binomial regression equations. The results revealed a strong correlation between different velocities and relative loads in BSS exercises (R^2^ = 0.880–0.964), with the MV-based model slightly outperforming the PV-based model (R^2^ difference of 0.06 and SEE difference of 0.02). Additionally, both MV and PV across all test loads demonstrated good reliability (ICC ≥ 0.801; CV ≤ 2.36%). Despite very strong correlations between the actual and predicted 1 RM across all models (R = 0.890–0.972), linear regression models and binomial regression models for the dominant leg consistently underestimated the actual 1 RM (p ≤ 0.035). Only the binomial regression models for the non-dominant leg accurately predicted the 1 RM (p ≥ 0.223). In conclusion, modeling using MV and binomial regression provides more reliable and accurate 1 RM predictions for BSS exercises. These findings confirm the utility of load-velocity relationships in predicting 1 RM for BSS exercises and suggest that practitioners should use MV and polynomial regression equations for predicting 1 RM in highly complex free-weight resistance exercises.

## Introduction

The one-repetition maximum (1 RM) is a key indicator for quantifying an individual's maximum strength level and is commonly used to determine the relative load (%1 RM) during training (Buckner et al., 2017). While the 1 RM can be directly assessed (McMaster et al., 2014), this method is time-intensive and requires athletes to bear extreme loads, which can lead to acute physical fatigue, injury, and potential disruptions in subsequent training sessions (Brechue and Mayhew, 2009; González-Badillo et al., 2011; Niewiadomski et al., 2008). An alternative to direct measurement is to use prediction equations based on the maximum number of repetitions performed at a given load (Banyard et al., 2018; Brzycki, 1993; Reilly et al., 2009). Although this approach reduces both the load and testing time (Hughes et al., 2019; Niewiadomski et al., 2008), it can still result in excessive fatigue and decreased strength in athletes (Banyard et al., 2018; Izquierdo et al., 2006, 2009). Additionally, the accuracy of these prediction equations can be compromised by factors such as the number of repetitions, training status, the exercise type, age, and sex (Jidovtseff et al., 2011; LeSuer et al., 1997; Whisenant et al., 2003). Therefore, a more suitable alternative that enables accurate 1-RM measurement while minimizing fatigue during testing is needed.

With advancements in technology, linear position transducers have become capable of accurately measuring barbell velocity during exercises (González-Badillo and Sánchez-Medina, 2010; Ruf et al., 2018). Recent studies have developed linear and polynomial regression models based on the inverse relationship between %1 RM and bar velocity to accurately predict 1 RM in various exercises, including the squat (Thompson et al., 2021), the bench press (Gonzalez-Badillo et al., 2010), the deadlift (Lake et al., 2017), and the prone bench pull (García-Ramos et al., 2019). This indirect prediction method allows for the estimation of 1 RM during the warm-up phase, assisting coaches in accurately quantifying and adjusting load intensity for the entire training session. However, it is important to note that different exercises, due to their unique movement patterns and primary muscle groups, exhibit different relationships between %1 RM and bar velocity (Fahs et al., 2019). Most existing studies have focused on the load-velocity relationship in bilateral exercises, while research on unilateral exercises, which are crucial in resistance training for improving unilateral limb strength and direction change ability, remains limited (Gonzalo-Skok et al., 2017; Speirs et al., 2016; Stern et al., 2020).

The Bulgarian split squat (BSS) is a common unilateral lower extremity exercise, where the foot of the non-working limb is supported on a bench behind the body while performing squats with a single leg. This exercise has been shown to reduce the support from the rear leg, thereby increasing the load on the front leg and introducing instability, which is essential for enhancing unilateral limb strength and stability (McCurdy, 2017). However, the complexity and instability of the BSS movement increase the risk of muscle injury during a 1-RM test (Mackey and Riemann, 2021). Consequently, it is necessary to establish a load-velocity relationship specific to the BSS to estimate %1 RM accurately. Current research has confirmed that linear position transducers could effectively measure movement velocity during BSS exercises (Chen et al., 2025a). However, to date, only two studies have explored the load-velocity relationship in BSS exercises. Liao et al. (2023) found that mean velocity (MV) was highly correlated with %1 RM in BSS exercises using both free weights and the Smith machine through linear regression models, but cross-prediction between these modalities was not possible. Another study by Rabal-Pelay et al. (2024) found a strong relationship between mean propulsion velocity (MPV) and %1 RM in BSS exercises through linear and polynomial regression models, with no significant differences observed between the dominant and non-dominant legs. However, these studies did not explore the differences in load-velocity relationships between various regression models and velocities in BSS exercises. To our knowledge, no reliable and accurate regression prediction method currently exists for predicting 1 RM in free-weight BSS exercises. Therefore, this study aimed to (a) investigate the load-velocity relationship in BSS exercises, (b) evaluate the reliability and validity of various 1 RM prediction models based on this relationship, and (c) determine the most reliable and effective regression model for predicting 1 RM in BSS exercises.

## Methods

### Participants

Twenty-seven men (mean ± standard deviation: age = 21.4 ± 1.3 years, body mass = 78.0 ± 9.5 kg, body height = 180.6 ± 7.8 cm, leg length = 86.8 ± 6.5 cm) participated in this study. All participants had over three years of strength-training experience (mean training duration = 6.5 ± 1.4 years) and were free from musculoskeletal disorders. They were instructed to refrain from strenuous activities for at least 24 hours before each testing session. Following a thorough explanation of the study's purpose and procedures, participants provided written informed consent. The study received approval from the Beijing Sport University ethics committee, Beijing, China (protocol code: No. 2023328H; approval date: 01 December 2023).

### Experimental Design

**Figure 1:**
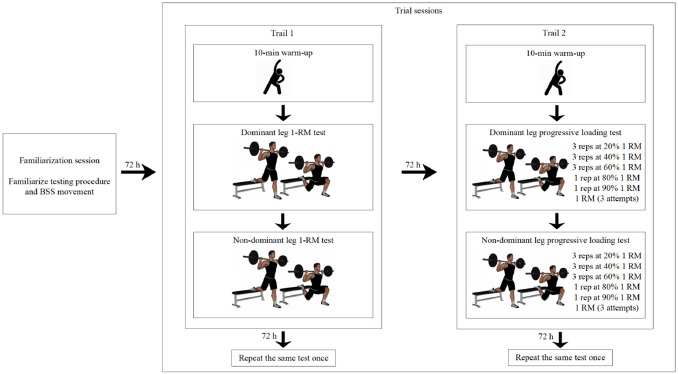
Experimental protocol. Trials 1 and 2 each comprised two 1-RM tests and progressive loading tests

Participants completed one familiarization session and two trial sessions (Trial 1 and Trial 2) (Figure 1). During the familiarization session, anthropometric data (age, body height, body mass, and leg length) were collected, and participants were introduced to the testing procedure and proper BSS technique. In Trial 1, participants underwent a 1-RM assessment to determine their actual 1 RM values. Trial 2 involved progressive loading tests (20–100% 1 RM), during which peak velocity and mean velocity were measured using a linear position transducer to establish their load- velocity relationships. To evaluate the reliability and validity of these relationships, participants repeated the experimental protocol in Trials 1 and 2 after a 72-h interval.

### Procedures

#### One Repetition Maximum Testing

Participants underwent two 1-RM tests using the same procedure during the Trial 1 session, with each test separated by a 72-h interval. A warm-up protocol was conducted before each test, consisting of 5 min of jogging, 3 min of dynamic stretching, and 2 min of joint mobility exercises. Following the warm-up, the 1-RM test was performed according to McCurdy et al. (2004). Initially, participants completed 5–10 repetitions with a light load, rested for 1 min, then completed 5 repetitions with a 10–20% increase in weight, followed by a 3–5-min rest interval. Next, the weight was increased by 20–30% to attempt a single repetition. If successful, the weight was increased by 10–20%; if unsuccessful, the weight was reduced by 5–10% before another attempt. This process continued until the 1 RM was determined, with a 3–5-min rest interval between each load. Participants were allowed to reach their 1 RM within 5 trials, including warm-up sets. To ensure adequate recovery, a minimum of 10 min separated the 1-RM tests of the dominant and non-dominant legs (Mausehund et al., 2019). The dominant leg was identified as the leg habitually used for kicking a ball (Mausehund et al., 2019; McCurdy et al., 2004, 2010). During all trials, participants positioned the barbell on the upper trapezius, with feet approximately shoulder-to-hip-width apart. The front foot was placed flat on the floor, and the instep of the rear foot rested on a bench behind the body, allowing both knees to remain slightly flexed. The distance between the bench and the front foot was set at 80% of the leg length (Aguilera-Castells et al., 2019), and the bench height was measured from the base of the patella to the floor (Mackey and Riemann, 2021). Participants flexed the hips and knees until the front thigh was parallel to the floor, then extended the knees and hips to return to the starting position. Two resistance-experienced spotters were present on either side of the bar to ensure safety throughout the test.

#### Load-Velocity Relationship Testing

Seventy-two hours after the 1-RM test, participants completed two load-velocity relationship tests following identical procedures. All loads were determined as percentages of the 1 RM from the most recent test. After performing the same warm-up protocol used for the 1-RM test, participants underwent incremental testing, which included 3 repetitions at 20%, 40%, and 60% of 1 RM, 1 repetition at 80% and 90% of 1 RM, and attempts at 100% of 1 RM. A maximum of three attempts was permitted to reach the 1 RM. Participants rested for 3 min between each load. During each repetition, the MV and peak velocity (PV) of the barbell during the concentric phase were measured using a Tendo linear position transducer (Tendo Sports Machines, Trencin, Slovak Republic). The device was positioned on the floor, with the retractable cord attached to one side of the bar and aligned perpendicularly. The reliability of the Tendo unit for velocity measurement has been validated previously (ICC > 0.91, SEM < 0.08 m•s^−1^) (Chéry and Ruf, 2019; Mangine et al., 2016; Spitz et al., 2019; Tsoukos et al., 2021).

### Statistical Analysis

Descriptive data are presented as means and standard deviations. The Shapiro-Wilk test was used to verify the normality of the sample data distribution (*p* > 0.05). Differences between the two 1 RM measurements were assessed using a paired sample *t*-test. The reliability of MV and PV at different %1 RM levels was evaluated using the intraclass correlation coefficient (ICC) and the coefficient of variation (CV) (Elsworthy et al., 2021). Linear regression and second-order polynomial regression equations for the dominant and non-dominant legs were established using multiple loads and velocities of the group data. To validate the equations, one set of data was used to fit the equations and a new set of data was used to predict the 1 RM, where the predicted 1 RM values were obtained by substituting the movement velocity at 1 RM of each subject into the fitted equations. The Pearson’s correlation coefficient (R), the coefficient of determination (R^2^), standard error of the estimate (SEE), and effect size (ES) were used to assess the validity of the predicted 1 RM against the actual measured values (García-Ramos et al., 2019). A paired sample *t*-test was used to analyze differences between the measured and predicted 1 RM values. Bland-Altman plots were used to assess the agreement and systematic bias between the measured and predicted 1 RMs. All analyses were conducted using 95% confidence intervals (95% CI), and a *p*-value of < 0.05 was considered statistically significant. All statistical analyses were performed using SPSS software (Version 24.0, Armonk, NY, USA).

## Results

In the two 1-RM testing sessions of Trial 1, the mean relative strength and 1RM values for the dominant leg were 1.32 ± 0.13 and 101.67 ± 6.20 kg in the first session, and 1.32 ± 0.13 and 102.00 ± 6.37 kg in the second session. For the non-dominant leg, the mean relative strength and 1 RM values were 1.27 ± 0.12 and 97.93 ± 6.28 kg in the first session, and 1.27 ± 0.13 and 98.15 ± 6.41 kg in the second session. There was no significant difference between the first and second tests for the 1 RM of the BSS (*p* = 0.08–0.161).

**Figure 2:**
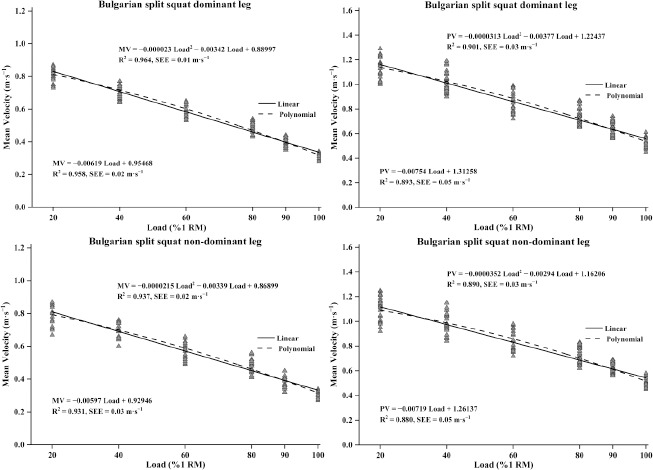
Relationship between the relative load (%1 RM) and different velocity variables obtained in the Bulgarian split squat dominant and non-dominant leg exercise. Solid line indicates linear regression and dashed line indicates polynomial regression; R^2^ indicates the coefficient of determination; SEE indicates standard error of the estimate

A strong relationship was found between MV and %1 RM for both the dominant leg (R^2^ = 0.958 for linear regression; R^2^ = 0.964 for polynomial regression) and the non-dominant leg (R^2^ = 0.931 for linear regression; R^2^ = 0.937 for polynomial regression). Similar findings were observed for PV and %1 RM in both the dominant leg (R^2^ = 0.893 for linear regression; R^2^ = 0.901 for polynomial regression) and the non-dominant leg (R^2^ = 0.880 for linear regression; R^2^ = 0.890 for polynomial regression). Notably, for each leg, R^2^ values for polynomial regression (R^2^ = 0.890–0.964) and MV (R^2^ = 0.931–0.964) were consistently higher than those for linear regression (R^2^ = 0.880–0.958) and PV (R^2^ = 0.880–0.901). In contrast, SEE values for polynomial regression (SEE = 0.01–0.03 m•s^−1^) and PV (SEE = 0.01–0.03 m•s^−1^) were consistently lower than those for linear regression (SEE = 0.02–0.05 m•s⁻^1^) and MV (SEE = 0.03–0.05 m•s^−1^). For detailed information, see Figure 2.

The mean values and test-retest reliability of MV and PV at 20%–100% of 1 RM for the two load-velocity relationship tests are summarized in Tables 1 and 2. The reliability of MV and PV at each %1 RM during the BSS exercise was very high. For the dominant leg, the ICC and CV for MV ranged from 0.812 to 0.919 and 1.07% to 1.46%, respectively. For PV, these values ranged from 0.811 to 0.936 and 1.48% to 1.82%, respectively. For the non-dominant leg, the ICC and CV for MV ranged from 0.801 to 0.934 and 1.20% to 1.98%, respectively. For PV, these values ranged from 0.827 to 0.931 and 1.70% to 2.36%, respectively. Furthermore, for both legs, the ICC for MV and PV increased with %1 RM, peaking at 90% 1 RM for MV and 80% 1 RM for PV. However, at 100% 1 RM, ICC values were lowest (Tables 1 and 2).

**Table 1:** Mean values and reliability of mean velocity and peak velocity for the Bulgarian split squat dominant leg at each %1 RM.

	Load (% 1 RM)	Dominant leg				
		Session 1 (m•s^−1^)	Session 2 (m•s^−1^)	Mean difference	ICC (95% CI)	CV (95% CI)
Mean velocity	20	0.81 ± 0.05	0.81 ± 0.05	−0.01 ± 0.03	0.856 (0.713, 0.932)	1.38 (1.08, 1.68)
	40	0.71 ± 0.05	0.71 ± 0.04	−0.01 ± 0.02	0.878 (0.743, 0.943)	1.29 (0.97, 1.61)
	60	0.60 ± 0.04	0.59 ± 0.04	0.00 ± 0.02	0.898 (0.790, 0.952)	1.46 (1.18, 1.73)
	80	0.49 ± 0.03	0.48 ± 0.03	0.00 ± 0.01	0.910 (0.814, 0.958)	1.39 (1.20, 1.57)
	90	0.40 ± 0.03	0.39 ± 0.02	0.00 ± 0.01	0.919 (0.826, 0.963)	1.07 (0.86, 1.29)
	100	0.31 ± 0.02	0.31 ± 0.01	−0.00 ± 0.01	0.812 (0.627, 0.910)	1.15 (0.84, 1.47)
Peak velocity	20	1.14 ± 0.07	1.14 ± 0.09	−0.01 ± 0.05	0.847 (0.694, 0.927)	1.82 (1.51, 2.13)
	40	1.01 ± 0.08	1.02 ± 0.08	−0.01 ± 0.04	0.894 (0.783, 0.950)	1.67 (1.36, 1.98)
	60	0.86 ± 0.08	0.87 ± 0.08	−0.01 ± 0.03	0.906 (0.805, 0.956)	1.79 (1.41, 2.17)
	80	0.74 ± 0.07	0.74 ± 0.07	−0.01 ± 0.02	0.936 (0.866, 0.970)	1.48 (1.14, 1.83)
	90	0.63 ± 0.05	0.64 ± 0.05	−0.01 ± 0.02	0.888 (0.760, 0.948)	1.79 (1.38, 2.20)
	100	0.52 ± 0.03	0.52 ± 0.04	−0.01 ± 0.02	0.811 (0.622, 0.910)	1.80 (1.51, 2.09)

ICC indicates the intraclass correlation coefficient; CV indicates the coefficient of variation; 95% CI indicates the 95% confidence interval

**Table 2:** Mean values and reliability of mean velocity and peak velocity for the Bulgarian split squat non-dominant leg at each %1 RM.

	Load (% 1 RM)	Non-dominant leg				
		Session 1 (m•s^−1^)	Session 2 (m•s^−1^)	Mean difference	ICC (95% CI)	CV (95% CI)
Mean velocity	20	0.79 ± 0.05	0.80 ± 0.05	−0.01 ± 0.03	0.834 (0.671, 0.920)	1.78 (1.47, 2.10)
	40	0.69 ± 0.05	0.69 ±0.05	−0.01 ± 0.02	0.867 (0.725, 0.937)	1.68 (1.44, 1.91)
	60	0.57 ± 0.05	0.58 ± 0.05	−0.01 ± 0.02	0.889 (0.771, 0.948)	1.98 (1.69, 2.26)
	80	0.49 ± 0.04	0.48 ± 0.03	0.00 ± 0.02	0.895 (0.782, 0.951)	1.57 (1.30, 1.84)
	90	0.39 ± 0.03	0.39 ± 0.03	0.00 ± 0.01	0.934 (0.854, 0.970)	1.20 (0.86, 1.54)
	100	0.31 ± 0.02	0.31 ± 0.02	−0.00 ± 0.01	0.801 (0.609, 0.904)	1.78 (1.28, 2.27)
Peak velocity	20	1.08 ± 0.08	1.10 ± 0.09	−0.01 ± 0.05	0.830 (0.633, 0.918)	2.36 (2.14, 2.59)
	40	0.96 ± 0.08	0.97 ± 0.09	−0.01 ± 0.04	0.886 (0.758, 0.947)	1.93 (1.57, 2.29)
	60	0.84 ± 0.08	0.85 ± 0.08	−0.01 ± 0.03	0.911 (0.815, 0.959)	1.76 (1.36, 2.16)
	80	0.72 ± 0.08	0.72 ± 0.07	−0.00 ± 0.03	0.931 (0.855, 0.968)	1.70 (1.35, 2.06)
	90	0.61 ± 0.05	0.62 ± 0.05	−0.00 ± 0.02	0.881 (0.758, 0.944)	1.73 (1.42, 2.04)
	100	0.51 ± 0.03	0.50 ± 0.04	−0.01 ± 0.02	0.827 (0.654, 0.918)	1.74 (1.42, 2.06)

ICC indicates the intraclass correlation coefficient; CV indicates the coefficient of variation; 95% CI indicates the 95% confidence interval

**Figure 3:**
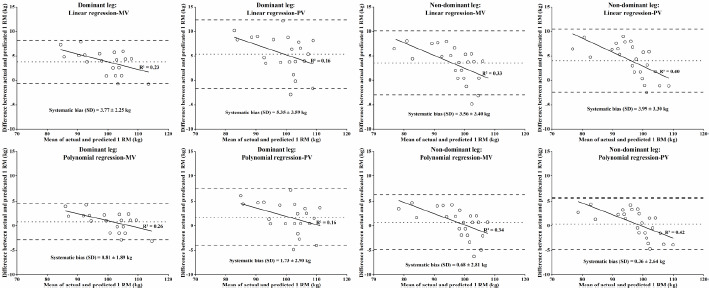
Bland-Altman plots showing the differences between the actual 1 RM and the predicted 1 RM from different predictive methods. Dotted lines indicate systematic bias, solid lines indicate regression lines, and dashed lines indicate 95% limits of agreement; R^2^ indicates the coefficient of determination

The validity analysis results for different prediction models are presented in Table 3. All linear regression models and binomial regression models for the dominant leg underestimated the actual 1 RM (*p* = 0.001–0.035). Only the binomial regression models for the non-dominant leg effectively predicted 1 RM (*p* = 0.223–0.488). Notably, the Pearson coefficients for the different 1 RM prediction models were very large or almost perfect (R = 0.890–0.972). Polynomial regression models consistently yielded higher Pearson correlation coefficients compared to linear regression models. This was also reflected in the effect size (0.05–0.25 for polynomial regression, indicating trivial or small effects; 0.48–0.75 for linear regression, indicating small or moderate effects) and standard error of estimation (0.34–0.52 kg for polynomial regression, 0.75–1.10 kg for linear regression). The Bland-Altman plots, shown in Figure 3, illustrate the agreement between predicted and actual 1 RM values. Polynomial regression models (systematic bias = 0.36–1.73 kg) demonstrated better agreement compared to linear regression models (systematic bias = 3.56–5.35 kg).

**Table 3:** Validity analyses of different methods to predict 1 RM of the Bulgarian split squat dominant and non-dominant legs.

	Predictive method	Actual 1 RM (kg)	Predicted 1 RM (kg)	*p*	R	ES (95% CI)	SEE (kg, 95% CI)
Dominant leg	Linear regression-MV	102.00 ± 6.37	98.23 ± 7.43	0.001*	0.958	0.54 (0.00, 1.09)	0.75 (0.60, 0.90)
	Polynomial regression-MV		101.19 ± 7.33	0.035*	0.972	0.12 (−0.42, 0.65)	0.34 (0.26, 0.41)
	Linear regression-PV		96.65 ± 7.77	0.001*	0.890	0.75 (0.20, 1.31)	1.10 (0.88, 1.31)
	Polynomial regression-PV		100.27 ± 7.52	0.005*	0.926	0.25 (−0.29, 0.78)	0.52 (0.38, 0.67)
Non-dominant leg	Linear regression-MV	98.15 ± 6.41	94.59 ± 8.29	0.001*	0.928	0.48 (−0.06, 1.02)	0.82 (0.64, 0.99)
	Polynomial regression-MV		97.47 ± 8.03	0.223	0.949	0.09 (−0.44, 0.63)	0.46 (0.34, 0.57)
	Linear regression-PV		94.16 ± 8.47	0.001*	0.939	0.53 (−0.01, 1.07)	0.85 (0.66, 1.04)
	Polynomial regression-PV		97.79 ± 8.11	0.488	0.961	0.05 (−0.48, 0.58)	0.44 (0.34, 0.53)

p indicates p-value, *significant difference compared to the actual 1 RM (p < 0.05); R indicates the Pearson’s correlation coefficient; ES indicates Cohen’s d effect size; SEE indicates standard error of estimation

## Discussion

This study evaluated the validity and reliability of various prediction methods based on the load-velocity relationship for estimating 1 RM during BSS exercises. The key findings were as follows: (a) a strong correlation was observed between mean velocity and peak velocity and the relative load during BSS exercises, (b) mean velocity and peak velocity demonstrated high reliability across all tested loads (20–100% 1 RM), (c) mean velocity with binomial regression provided better prediction accuracy compared to peak velocity with linear regression, and (d) only binomial regression models for the non-dominant leg effectively predicted 1 RM, while other models tended to underestimate actual 1 RM values. Overall, these results suggest that mean velocity and peak velocity can be used to estimate the %1 RM in BSS exercises, with binomial regression using mean velocity being the most accurate method for predicting 1 RM in these exercises.

The load-velocity relationship is widely used to estimate exercise intensity, helping coaches adjust athletes' training loads effectively throughout the day. This study confirmed a strong load-velocity relationship between different velocities and relative loads during BSS exercises at 20–100% 1 RM (R^2^ = 0.880–0.964). This finding aligns with previous research demonstrating a robust relationship between relative load and movement velocity in exercises such as Bulgarian split squats (Liao et al., 2023; Rabal-Pelay et al., 2024), back squats (Conceição et al., 2016; Martínez-Cava et al., 2019; Sánchez-Medina et al., 2017), deadlifts (Benavides-Ubric et al., 2020; Morán-Navarro et al., 2021), bench presses (García-Ramos et al., 2021; González-Badillo and Sánchez-Medina, 2010; Pestaña-Melero et al., 2018), prone pull-ups (Muñoz-López et al., 2017), leg presses (Conceição et al., 2016), half-squats (Conceição et al., 2016; Martínez-Cava et al., 2019; Pérez-Castilla et al., 2020), and military presses (Balsalobre-Fernández et al., 2018). Notably, the load-velocity relationships observed in this study were weaker than those reported by Rabal-Pelay et al. (2024) (R^2^ = 0.94–0.98), but stronger than those found by Liao et al. (2023) (R^2^ = 0.85). This may be due to the fact that Rabal-Pelay et al. (2024) included a pause between the eccentric and concentric phases to eliminate the influence of muscle elastic potential energy during the stretch-shortening cycle. Although this helped reduce the test errors (Conceição et al., 2016; Pallarés et al., 2014), it also affected the ecological validity of athletes (Banyard et al., 2017, 2018; Conceição et al., 2016), because athletes usually need to fully utilize the stretch-shortening cycle during various resistance and technical training (such as weightlifting, running, jumping) to generate greater force and velocity, so as to obtain better training results (Banyard et al., 2017, 2018). Furthermore, compared to the study by Liao et al. (2023), this investigation included two additional 1-RM tests for BSS exercises prior to the load-velocity relationship tests. Previous studies have shown that two consecutive 1-RM tests performed on separate days can lead to increases in muscle strength, which is thought to be due to neuromuscular adaptation (Dias et al., 2005; Levinger et al., 2009; Ritti-Dias et al., 2011). Although there was no significant difference between the two actual 1 RM values in this study, the strength test could also have enabled subjects to obtain improvements in neuromuscular adaptation, resulting in enhanced muscle coordination and postural control (Carroll et al., 2001; Wang et al., 2024), which may be beneficial to reduce the test errors.

This study also found that the goodness of fit for the load-velocity relationship using binomial regression was slightly better than when using linear regression (R^2^ difference of 0.008, SEE difference of 0.02). This finding supports previous research indicating that second-order polynomials provide a marginally better fit than linear functions for the relationship between %1 RM and velocity (R^2^ difference of 0.005). Although Rabal-Pelay et al. (2024) reported no significant difference in R^2^ between linear and binomial regression equations, R^2^ value for binomial regression was slightly higher than for linear regression (R^2^ difference of 0.002). In terms of velocity, mean velocity was better fitted than peak velocity (R^2^ difference of 0.06). This may be because mean velocity describes the average velocity of the barbell throughout the entire concentric phase, while peak velocity reflects the maximum instantaneous velocity achieved during the lift, which is greatly influenced by the stretch-shortening cycle. Since this study did not eliminate the stretch-shortening cycle effect during the BSS movement, the variability in peak velocity during measurement increased, thereby reducing the association between peak velocity and the relative load.

Test-retest reliability indicates the stability of the load-velocity curve, typically assessed using the intraclass correlation coefficient and the coefficient of variation (Hopkins, 2000). This study evaluated the test-retest reliability of two velocity indices for both the dominant and non-dominant legs and found that both mean velocity and peak velocity demonstrated high reliability across 20–100% of 1 RM (ICC ≥ 0.801; CV ≤ 2.36%). Previous studies have reported poor reliability for mean velocity at 20% and 90% of 1 RM (ICC = 0.25–0.38, CV = 7.2–14.2%) (Hughes et al., 2019), as well as at 100% of 1 RM (ICC = 0.42; CV = 22.5%; ICC = 0.55; CV = 19.4%) (Banyard et al., 2017, 2018). Although this study found that mean velocity and peak velocity were highly reliable at both 20% and 100% of 1 RM, their reliability was lower compared to other loads (40–90% of 1 RM), particularly at 100% of 1 RM. This may be due to the increased energy required to maintain postural stability during single-leg landing in BSS exercises. At extreme loads or velocities, participants experience greater instability, demanding more from the support leg strength and trunk stability, which can lead to issues such as heel lifting, trunk swaying, and changes in the barbell's movement trajectory. Thus, the reliability of the load-velocity relationship may be influenced by the participants’ movement patterns and strength levels.

The validity of the load-velocity relationship is crucial for applying velocity-based prediction methods in exercise practice, as it is affected by the types of velocity measured and the prediction models used. To identify the most effective method for predicting 1 RM in BSS exercises, we fitted eight different prediction models and observed a strong correlation between predicted and actual 1 RM across all models (R = 0.859–0.980). However, this study found that general linear regression models were not effective in predicting 1 RM in BSS exercises, which aligns with the findings of recent research (Liao et al., 2023). In contrast, polynomial regression models provided more accurate 1 RM predictions, as evidenced by trivial and small effect sizes and lower standard errors of estimate. This result was anticipated, as Thompson et al. (2021) also reported that binomial models outperformed linear regression models in predicting 1 RM in back squat exercises. They suggested that lower body movements were more complex, necessitating the use of more sophisticated prediction models for lower limb exercises. As a multi-joint exercise, the BSS requires coordination across the entire body, and single-leg support further adds complexity to the movement. Therefore, for highly complex free-weight exercises, a more intricate polynomial regression model should be used to predict 1 RM rather than a simple linear regression model.

However, the binomial prediction models, such as linear models, underestimated the actual 1 RM for the dominant leg. Only the binomial models for the non-dominant leg accurately predicted 1 RM. This discrepancy may be due to the cumulative nerve and muscle fatigue from prolonged testing, which caused subjects to produce lower movement velocities at the same load, leading to underestimation by the load-velocity relationships. The testing sequence also differed between the dominant and non-dominant legs, resulting in varying loading stimuli. The dominant leg, tested first, bore more weight initially and might induce more significant muscle adaptation and fatigue accumulation. Despite resting between tests, individual recovery rates varied, affecting the muscle condition of the dominant leg at each test. As a result, the load-velocity relationships for the dominant leg underestimated the actual 1 RM. Bland-Altman plots further assessed the variation and error between predicted and measured 1 RM values for different models (Giavarina, 2015). These plots revealed that binomial regression models predicted 1 RM with smaller systematic bias and higher agreement with the actual 1 RM. In summary, the mean velocity and binomial regression models provided more reliable and valid 1 RM predictions than the peak velocity and linear regression models.

Although this study demonstrated that the load-velocity relationship could effectively predict the 1 RM of the BSS exercise, several limitations remain. Since the BSS is a unilateral lower limb exercise, both dominant and non-dominant legs must be tested within the same session, inevitably increasing neuromuscular fatigue. Despite an interval between testing the dominant and non-dominant legs, fatigue accumulation may still compromise test reliability and validity. Recent study demonstrated that the two-point method (using two loads) could serve as an effective and fatigue-free alternative to the multi-point approach when establishing the load-velocity relationship in squat exercises (Chen et al., 2025b), while its applicability to BSS exercises remained to be elucidated. Furthermore, this study established only general load-velocity relationships, whereas previous research (Liao et al., 2023) has shown that individual load-velocity relationships based on mean velocity offer more accurate 1 RM predictions for BSS exercises. However, the reliability and validity of load-velocity relationships derived from other velocity indicators remain unclear. Future research should explore whether individual load-velocity relationships, developed using different models and velocity indicators, can reliably and accurately predict 1 RM in BSS exercises.

## Conclusions

In conclusion, barbell velocity was found to be an effective and reliable method for predicting the % 1 RM of the Bulgarian split squat exercises. This suggests that coaches can monitor and adjust the training load of Bulgarian split squat exercises based on movement velocity, allowing for timely modifications to the daily training plan. Additionally, mean velocity and binomial regression models provided more reliable and accurate 1 RM predictions compared to peak velocity and linear regression models. Therefore, we do not recommend using linear regression models to establish load-velocity relationships for complex exercises such as the Bulgarian split squat. Finally, the conclusions of this study are confined to young males with long-term resistance training experience, and caution should be exercised when generalizing these results directly to other populations.
